# Complement C3a receptor inactivation attenuates retinal degeneration induced by oxidative damage

**DOI:** 10.3389/fnins.2022.951491

**Published:** 2022-08-30

**Authors:** Shaojun Wang, Lu Du, Shunzong Yuan, Guang-Hua Peng

**Affiliations:** ^1^Laboratory of Visual Cell Differentiation and Regulation, Basic Medical College, Zhengzhou University, Zhengzhou, China; ^2^Senior Department of Ophthalmology, Chinese People’s Liberation Army (PLA) General Hospital, Beijing, China; ^3^Department of Lymphoma, Head and Neck Cancer, The Fifth Medical Center, Chinese People’s Liberation Army (PLA) General Hospital (Former 307th Hospital of the PLA), Beijing, China; ^4^Department of Pathophysiology, Basic Medical College, Zhengzhou University, Zhengzhou, China

**Keywords:** complement C3, C3a receptor (C3ar1), retinal degeneration, apoptosis, microglia

## Abstract

Retinal degeneration causes vision loss and threatens the health of elderly individuals worldwide. Evidence indicates that the activation of the complement system is associated with retinal degeneration. However, the mechanism of complement signaling in retinal degeneration needs to be further studied. In this study, we show that the expression of C3 and C3a receptor (C3ar1) is positively associated with the inflammatory response and retinal degeneration. Genetic deletion of C3 and pharmacological inhibition of C3ar1 resulted in the alleviation of neuroinflammation, prevention of photoreceptor cell apoptosis and restoration of visual function. RNA sequencing (RNA-seq) identified a C3ar1-dependent network shown to regulate microglial activation and astrocyte gliosis formation. Mechanistically, we found that STAT3 functioned downstream of the C3-C3ar1 pathway and that the C3ar1-STAT3 pathway functionally mediated the immune response and photoreceptor cell degeneration in response to oxidative stress. These findings reveal an important role of C3ar1 in oxidative-induced retinal degeneration and suggest that intervention of the C3ar1 pathway may alleviate retinal degeneration.

## Introduction

Retinal degeneration threatens the vision of elderly people around the world due to the gradual degeneration of photoreceptor cells that leads to complete blindness (Handa et al., 2019). Unfortunately, no effective treatments are available for retinal degeneration (Wong et al., 2014). Retinal degeneration is often accompanied by microglial cell activation and elevated levels of inflammatory cytokines as well as photoreceptor cell loss and retinal structure destruction (Ambati and Fowler, 2012; Menon et al., 2019; Voigt et al., 2019; Mulfaul et al., 2020; Orozco et al., 2020). These studies strongly suggest that the innate immune response plays an important role in retinal degeneration.

The complement system is a major component of the innate immune system and plays a key role in retinal development and homeostasis (Ricklin et al., 2016; Liszewski et al., 2017; Akhtar-Schafer et al., 2018). Studies have found elevated expression and deposition of C3 in damaged retinas (Chi et al., 2010; Stephan et al., 2012; Ramos de Carvalho et al., 2013; Linetsky et al., 2018). C3 activation is crucial for triggering immune responses causing retinal diseases (van Lookeren Campagne et al., 2016; Mohlin et al., 2017; Enzbrenner et al., 2021). During the activation of the classical complement pathway, C3 is cleaved into two fragments, C3a and C3b, which bind to the corresponding receptors to thereby regulate the innate immune response. The C3b-CR3 pathway has been demonstrated to be critical for the clearance of debris by microglia after CNS injury (Norris et al., 2018). In the rd10 retina, both C3 and CR3 mediate the inflammatory response and the microglial phagocytosis of apoptotic photoreceptors (Silverman et al., 2019).

Studies have also shown that the C3b-CR3 signaling pathway is involved in development, neuropathology and the inflammatory response (Litvinchuk et al., 2018). In a mouse model of AD and epilepsy, the C3a-C3ar1 axis was found to be involved in microglial activation and astrocyte gliosis formation (Lian et al., 2016; Wei et al., 2021). In addition, blockade of the C3a/C3aR axis alleviates severe acute pancreatitis-induced intestinal barrier injury by repressing inflammatory cytokines (Ye et al., 2020). Complement is also involved in orchestrating regeneration progression, as the C3ar1 signaling pathway facilitates skeletal muscle regeneration by regulating monocyte function and trafficking (Zhang et al., 2017). C3ar1 also plays an important role in chick retinal development and regeneration (Grajales-Esquivel et al., 2017).

Although C3ar1 is involved in retinal development and neuropathy, the role of the C3-C3ar1 axis in retinal degeneration is less understood. In our study, we assessed the roles of complement C3, its receptor C3aR and the downstream effector STAT3 in the regulation of retinal degeneration in a model of sodium iodate-induced oxidative stress using various techniques, such as gene knockout, immunofluorescence, RNA sequencing (RNA-seq) and functional assays. Furthermore, we identified that the C3a-C3ar1-STAT3 axis plays an important role in mediating microglial activation and photoreceptor cell injury.

## Materials and methods

### Animals

All studies were carried out on 8-week-old wild-type or C3-knockout (C3-KO) C57BL/6J mice. The C3-KO C57BL/6J mice were provided by Professor Yusen Zhou (State Key Laboratory of Pathogen and Biosecurity, Beijing Institute of Microbiology and Epidemiology, Beijing 100071, China). All animals were housed in a pathogen-free, temperature-controlled animal facility on a 12/12-h light/dark cycle and fed standard food and water *ad libitum*. All of the animal protocols were approved by the Institutional Animal Care and Use Committee of the General Hospital of the Chinese People’s Liberation Army and Zhengzhou University and were performed in accordance with the National Institutes of Health Guidelines for the Care and Use of Laboratory Animals (Id number:2020-ky-67). All efforts were made to reduce the number of animals used and minimize the suffering caused by experimental procedures.

### Treatment with sodium iodate and tissue collection

We selected sodium iodate (NaIO_3_) (Sigma–Aldrich, United States) for the establishment of the retinal degeneration model according to a previous publication (Xiao et al., 2017). Briefly, mice in the experimental groups were administered a single dose of 35 mg/kg NaIO_3_ dissolved in saline *via* femoral vein injection. The mice in the control group were administered an equivalent volume of saline. The NaIO_3_-treated and control mice (*n* = 6 per group) were examined by electroretinography (ERG) and multifocal electroretinography (mfERG), and their eyeballs were then collected from days 1 to 28 by enucleating the mice and immersing the eyeballs in 4% paraformaldehyde in 0.1 M phosphate buffer.

### Histology and immunofluorescence

After fixation for 24 h, the anterior section of the eyes was dissected, and the remaining eye cup was dehydrated and then embedded in paraffin wax. Sections (at 5 μm) were cut on a microtome (Shandon AS325; Thermo Scientific, United States). All histologic analyses of outer nuclear layer (ONL) thickness were performed using retinal sections cut along the parasagittal plane (super inferior) as described previously (Wang et al., 2014). These sections also included the ocular nerve head to maintain regional consistency between replicates and groups. H&E staining was performed, and images were captured with a microscope (Olympus, Japan). For each section, 18 measurements spaced 200 μm apart were made to analyze ONL damage. Three measurements were made per sample and averaged.

For immunostaining, sections were dewaxed, subjected to antigen retrieval and blocked with 10% goat serum (Sigma–Aldrich Corp.) in phosphate-buffered saline containing 0.2% Triton X-100 (Sigma–Aldrich Corp.) for 1 h before being incubated with primary antibodies in a humidity chamber overnight at 40°C. The primary antibodies were as follows: anti-complement C3, 1:300; anti-Ibal, 1:300; anti-rhodopsin, 1:600; anti-C3aR, 1:300; anti-RPE65, 1:300 and anti-pSTAT3, 1:300. All antibodies were from purchased Abcam. After washing and incubation for 1 h at room temperature with secondary antibodies, the sections were counterstained with ProLong Gold with DAPI (Invitrogen) to reveal cell nuclei. Images were obtained using an Olympus FV3000 confocal microscope and were acquired at the corresponding histologically defined areas of the sections. Schematic of the retina with a sample location was shown in Supplementary Figure 1. All images in each individual experiment were acquired with a fixed detection gain. Images were processed and semiquantified by using ImageJ.

### Electroretinography

ERG was performed using the Espion E3 console in conjunction with the ColorDome (Diagnosys LLC). ERG experiments were carried out on C57B6 wild-type or C3-KO mice at 1 week post injection. In brief, the mice were dark-adapted the night before the recordings were performed. The animals were anesthetized by a subcutaneous injection of xylazine (15 mg/kg) and ketamine (110 mg/kg). The pupils of the mice were dilated using 1% tropicamide, and the animals were positioned on a water warming pad to prevent hypothermia. For each animal, only the right eye was examined. Active gold electrodes were placed on the right eye cornea as the recording electrodes. The reference and ground electrodes were placed subcutaneously in the mid-frontal areas of the head and tail, respectively. Light stimulation was applied at a density of 0.5 log (cd⋅s/m^2^). The amplitudes of a- and b-waves were recorded and processed using a RETI-Port device (Roland Consult). All procedures were performed in a dark room under a dim red safety light.

### TdT-UTP nick end labeling staining

TdT-UTP nick end labeling (TUNEL) assays were performed with a one-step TUNEL kit according to the manufacturer’s instructions (Beyond, Shanghai). Paraffin-embedded sections and cells grown in 24-well plates treated with NaIO_3_ were fixed. Briefly, the cells were permeabilized with 0.1% Triton X-100 for 10 min at room temperature, followed by TUNEL staining for 1 h at 37°C. The FITC-labeled TUNEL-positive cells were imaged under a fluorescence microscope at 488 nm excitation and 530 nm emission wavelengths. The cells with green fluorescence were defined as apoptotic cells.

### Application of drugs

A C3aR antagonist (SB290157) and STAT3 inhibitor (SH-4–54) were purchased from Selleck (S8931 and S7337) and dissolved according to the manufacturer’s instructions. Mice were pretreated with SB290157 (10 mg/kg) or SH-4-54 (10 mg/kg) 3 days before the NaIO_3_ injection and then three treated times a week *via* i.p. injection for 1 week. After the ERG assay, the mice were sacrificed, and retinal tissue was collected for immunoassay and real-time PCR assays.

### Ribonucleic acid extraction and real-time polymerase chain reaction

Total RNA was extracted, and a real-time PCR assay was conducted as previously reported (Wang et al., 2019). The primer sequences are listed below:

**Table:** 

Gene	F	R
**mC3**	GAAGTACCTCATGT GGGGCC	CAGTTGGGACAA CCATAAACC
**mC3aR**	GGAAGCTGTGATG TCCTGG	CACACATCTGTA CTCATATTGT
**mStat3**	CAGAAAGTGTCCTA CAAGGGCG	CGTTGTTAGACT CCTCCATGTTC
**mRhodopsin**	CCCTTCTCCAACGT CACAGG	GTAGAGCGTGA GGAAGTTGATG
**mRecoverin**	CAATGGGACCATCA GCAAA	CCTCAGGCTTG ATCATTTTGA
**mTNFα**	CCCTCACACTCAGATC ATCTTCT	GCTACGACGT GGGCTACAG
**mIL1β**	GCAACTGTTCCTGAA CTCAACT	ATCTTTTGGG GTCCGTCAACT
**mIL6**	TAGTCCTTCCTACCC CAATTTCC	TTGGTCCTTAG CCACTCCTTC
**mβ –actin1**	CGAGAAGATGACC CAGATCATGTT	CCTCGTAGAT GGGCACAGTGT
		

### Ribonucleic acid-seq and data analysis

First, retinal tissues were collected from normal controls and wild-type mice at 7 days after NaIO_3_ induction, and total RNA was then extracted. The RNA-seq analysis was performed on the Illumina NovaSeq6000 platform at a depth of 60 million read pairs per sample. Three mice from each group (total of 6 mice) were analyzed. Raw reads were first aligned to the Mus musculus genome (UCSC mm10) using HISAT2 with default parameters. Then, the htseq-count function of HTSeq was used to determine the number of aligned reads that fell under the exons of the gene (union of all the exons of the gene) to present the expression of each gene. We identified differentially expressed genes (DEGs) by using the DESeq2 package in the R environment (Love et al., 2014). Finally, Gene Ontology (GO) analysis was performed.

### Statistical analysis

Statistical analysis of the two groups was performed using 2-tailed paired Student’s *t*-tests assuming equal variance. The results are expressed as the mean ± SEM deviation. Differences were considered significant at *P* < 0.05.

## Results

### Complement C3 and C3ar1 expression correlates with retinal degeneration

**FIGURE 1:**
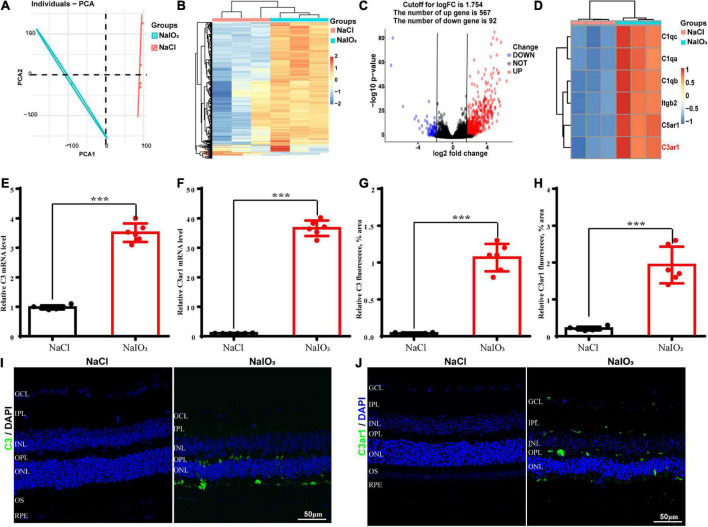
Upregulation of C3 and C3aR in the NaIO_3_ mouse retinal degeneration model. **(A)** PCA graph showing the distribution of RNA-seq samples between the control and NaIO_3_-treated groups. **(B)** Unsupervised clustering of the top 500 genes expressed above background levels demonstrated similar patterns of gene expression between the control and NaIO_3_-treated groups. **(C)** Volcano plots highlighting the distributions of DEGs between the control and NaIO_3_-treated groups. **(D)** Some differentially expressed complement component genes between the control and NaIO_3–_treated groups are shown as a heatmap. **(E,F)** The mRNA levels of C3 and C3aR in the control and NaIO_3_-treated groups were analyzed by real-time PCR. **(G–J)** Immunofluorescence staining and semiquantified fluorescence levels of C3 (green) and C3ar1 in saline control- and NaIO_3_-treated retinas on day 7; nuclei were counterstained with DAPI (blue) (Bars, 50 μm). The data are expressed as the mean ± SEM ***P < 0.001, *n* = 6, unpaired *t*-test, two-tailed.

To further understand the molecular mechanisms underlying oxidative stress-induced retinal degeneration, we performed RNA-seq analyses of retinal tissues from 6- to 8-week-old wild-type C57BL/6J normal control and NaIO_3_-treated C57BL/6J mice. Using principal component analysis (PCA), we identified 659 DEGs (adjusted *p* < 0.01, cutoff for LogFC is 1.754) in mice treated with NaIO_3_ compared to the normal controls; 567 genes were upregulated and 92 were downregulated (Figures 1A–C). We found that the expression levels of complement genes were changed after NaIO_3_-induced retinal degeneration. Several complement components (C1qc, C1qa, C1qb, Itgb2, C5ar1, and C3ar1) had increased expression (Figure 1D). The real-time PCR results confirmed that the levels of C3 and C3ar1 were increased after NaIO_3_ treatment (Figures 1E,F). Immunofluorescence staining revealed prominent C3 and C3ar1 protein expression in the injured retinas but not in those of the normal control mice (Figures 1G–J), which provided direct evidence of oxidative stress-induced complement activation in mouse retinal tissues.

### C3ar1 controls the immune interaction network

**FIGURE 2:**
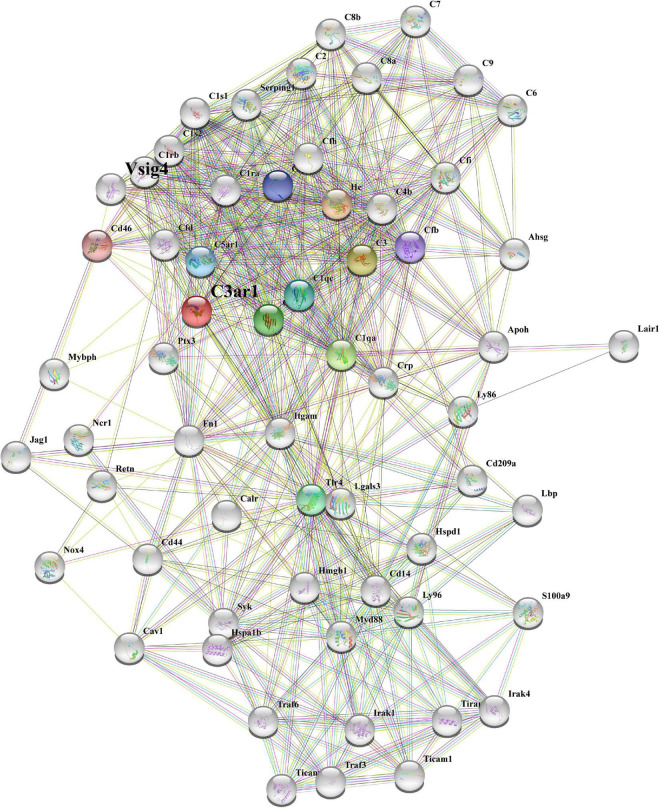
STRING network analysis reveals a C3ar1-centered immune interaction network. STRING network analysis of the C3ar1-related protein interaction network. Network nodes represent proteins. Edges represent protein–protein associations. Number of nodes: 11, number of edges: 37, average node degree: 6.73, average local clustering coefficient: 0.849, expected number of edges: 11, PPI enrichment *p*-value: 3.45e-10.

To better determine the role of C3ar1 in retinal degeneration, we searched the STRING database and constructed the protein interaction network centered on C3ar1. Based on coexpression scores determined by RNA expression patterns and on protein coregulation determined by Proteome HD, the network included a group of genes associated with retinal degeneration that are involved in the positive regulation of apoptotic cell clearance, synapse pruning, microglial activation, immune responses and complement pathway activation (Figure 2). For example, V-set and immunoglobulin domain-containing protein 4 (VSIG4), which is a strong negative regulator of T-cell proliferation and IL2 production, strongly interacts and coexpresses with C3ar1. The P2X purinoceptor 7 (P2RX7) receptor, a ligand-gated ion channel that responds to ATP binding, is responsible for the ATP-dependent lysis of macrophages through the formation of membrane pores permeable to large molecules. The P2RX7 receptor functions in both fast synaptic transmission and the ATP-mediated lysis of antigen-presenting cells. Together, these bioinformatics data suggest that the activation of the C3a-C3ar1 signaling pathway contribute to retinal degeneration through regulation of the inflammatory response and immune cell activation.

### Knockout of C3 and C3ar1 inactivation reduce microglial activation and the inflammatory response in mice with retinal degeneration

**FIGURE 3:**
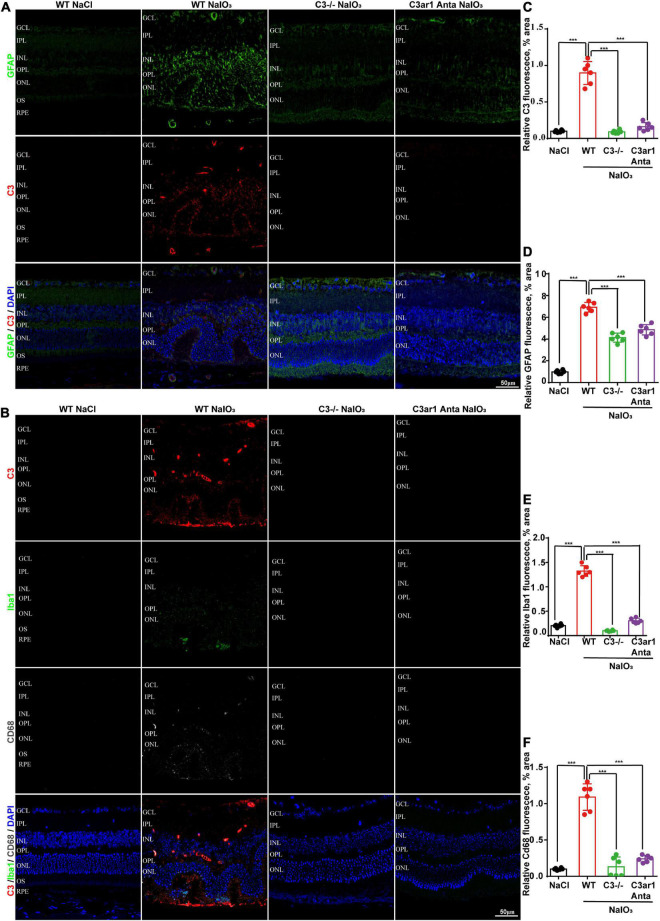
Genetic deletion of C3 and inactivation of C3ar1 attenuate reactive gliosis and microglial infiltration. **(A)** Representative GFAP and C3 multicolor immunofluorescence staining in the retinas of saline control- and NaIO3-treated WT, C3-KO and C3ar1-inhibited mice. Scale bar: 50 mm. **(B)** Representative C3, Iba1 and CD68 multicolor immunofluorescence staining in the retinas of saline control- and NaIO3-treated WT, C3-KO and C3ar1-inhibited mice. Scale bar: 50 mm. **(C–F)** Quantification of C3, GFAP, Iba1 and CD68 immunoreactivities. The data represent the mean ± SEM, *n* = 6. ***p < 0.001.

**FIGURE 4:**
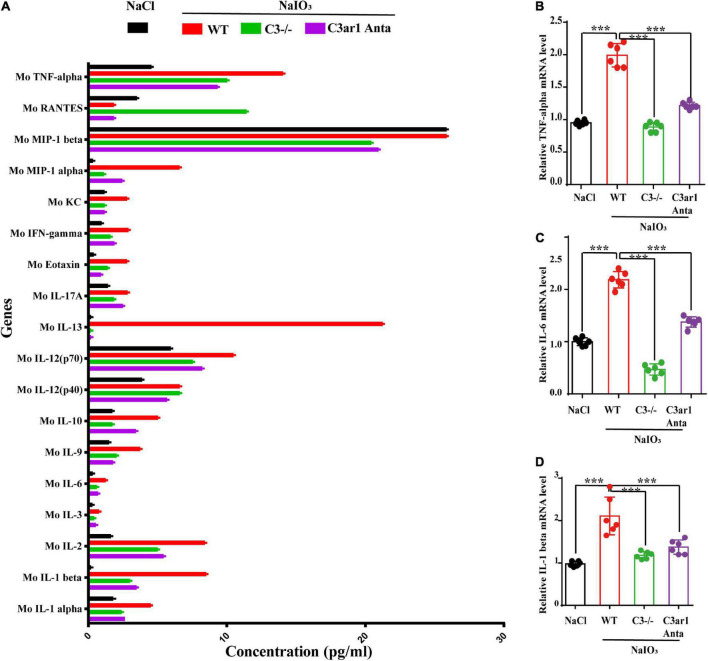
Cytokine retinal array analysis reveals high levels of proinflammatory factors. **(A)** Clustered column graph of the retinal cytokine profiles of saline control- and NaIO3-treated WT, C3-KO and C3ar1-inhibited mice obtained by using the Integral Molecular@QAM-INF-1 kit. **(B–D)** The retinal Il-6, TNF-α and TGF-β2 mRNA expression levels were determined by qRT–PCR. Each bar corresponds to the mean ± *SD*. ***p < 0.001.

The immune response plays an important role in photoreceptor cell degeneration (Murakami et al., 2019). To investigate the role of the C3a-C3ar1 pathway in immune regulation and retinal degeneration, immunostaining with anti-GFAP and anti-Iba1 antibodies revealed marked increases in the fluorescence intensities of GFAP and Iba1 in retinas on day 7 after NaIO_3_ stimulation (Figure 3). Strikingly, C3 deficiency and C3ar1 inhibition nearly completely normalized the GFAP and Iba1 immunoreactivity levels (Figure 3). To identify the retinal locus of C3 on day 7 after NaIO_3_ induction, we performed fluorescence immunostaining with multiple antibodies, including anti-GFAP, anti-Iba1, anti-CD68 and anti-C3 antibodies (Figures 3A,B). C3 colocalized predominantly with Iba1^+^ cells, indicating that microglia were activation during photoreceptor cell degeneration. Triple immunostaining of CD68, C3 and Iba1 revealed the characteristics of microglial phenotypes. Oxidative stress induced an increase in the number of CD68-positive phagocytic microglia, while C3 deficiency and C3ar1 inhibition drastically decreased the number of microglial phagocytic phenotypes. Microglia and Müller glia activation is associated with an increased production of proinflammatory cytokines. To elucidate the characteristics of the inflammatory cytokines, we performed cytokine array assays using QAM-INF-1, revealing increased levels of proinflammatory factors, including TNFα, IL1, IL6, MIP1α, IFNγ, eotaxin, and IL13 (Figure 4A). C3 deficiency and C3ar1 inhibition obviously downregulated the expression of these inflammatory factors. Using real-time PCR, we confirmed that the levels of TNFα, IL1, and IL6 were increased after the application of NaIO_3_, and C3 deficiency and C3ar1 inhibition obviously downregulated the mRNA levels of these genes (Figures 4B–D). These results support that C3aR regulates astrocyte and microglial reactivity and the production of proinflammatory cytokines in retinas with oxidative stress injury.

### Knockout of C3 and C3ar1 inactivation reduce photoreceptor cell apoptosis and restore visual function in mice with retinal degeneration

**FIGURE 5:**
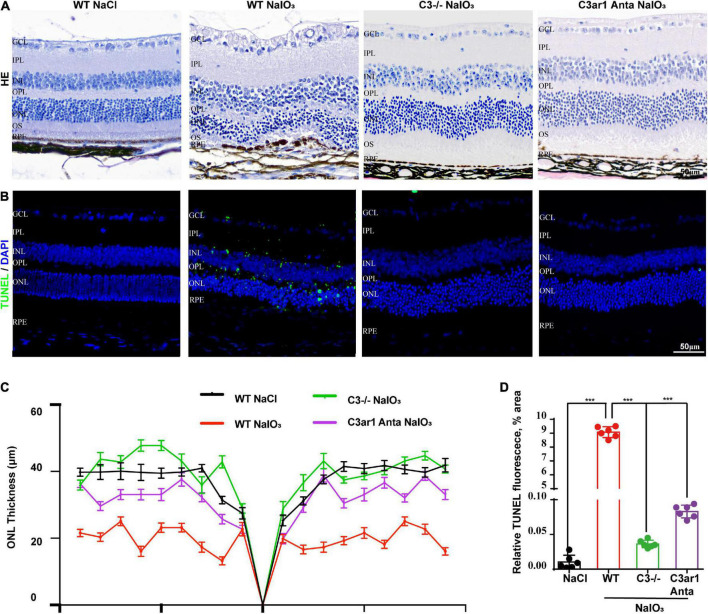
Genetic deletion of C3 and inactivation of C3ar1 protects the neural retina structure. **(A)** H&E staining of retinas of saline control- and NaIO3-treated WT, C3-KO and C3ar1-inhibited mice. **(B)** TUNEL immunofluorescence staining of retinas of saline control- and NaIO_3_-treated WT, C3-KO and C3ar1-inhibited mice. **(C)** Quantification of ONL thickness in the retinas of saline control- and NaIO3-treated WT, C3-KO, and C3ar1-inhibited mice. **(D)** Immunofluorescence staining and quantification of TUNEL in retinas of saline control- and NaIO3-treated WT, C3-KO, and C3ar1 inhibition mice.

**FIGURE 6:**
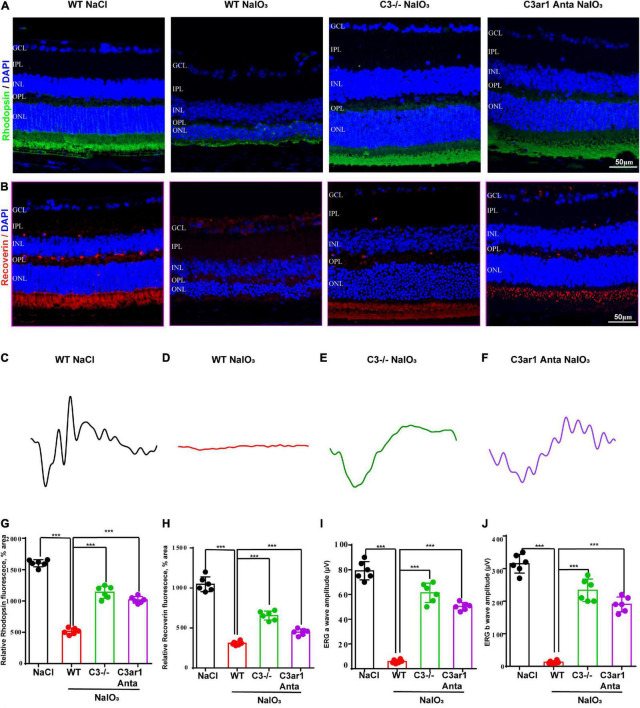
Genetic deletion of C3 and inactivation of C3ar1 rescues the retinal function of wild-type mice after NaIO_3_ induction. **(A,B)** Immunofluorescence staining of rhodopsin and recoverin in the retinas of saline control- and NaIO3-treated WT, C3-KO and C3ar1-inhibited mice. **(C–F)** ERG analysis of retinal function in saline control- and NaIO3-treated WT, C3-KO and C3ar1-inhibited mice (dMax 3.0). **(G,H)** Quantification of rhodopsin and recoverin in the retinas of saline control- and NaIO3-treated WT, C3-KO and C3ar1-inhibited mice. **(I,J)** Bar plot of the amplitudes of a and b waves in saline control- and NaIO3 treated WT, C3-KO and C3ar1-inhibited mice (mean ± SEM, *n* = 6. ***p < 0.001).

The above studies demonstrated the prominent role of C3a-C3ar1 in immune regulation. We also assessed the role of C3a-C3ar1 signaling in photoreceptor cell degeneration. H&E staining revealed a significant reduction in the ONL thickness on day 7 after NaIO_3_ induction compared to that in the control group. In contrast, the ONL thickness in mice with C3 deficiency and C3ar1 inhibition did not significantly differ from that in the controls on day 7 (Figures 5A,C). Furthermore, TUNEL staining showed that the apoptosis of photoreceptor cells was significantly reduced in the C3-KO and C3ar1-inhibited groups on day 7 (Figures 5B,D). These results indicated that C3a-C3ar1 played an important role in NaIO_3_-induced retinal photoreceptor cell degeneration and that the inactivation of C3 restored retinal function. Remarkably, the ERG a-wave and b-wave amplitudes were significantly decreased in wild-type mice on day 7 after NaIO_3_ induction but increased in C3-deficient and C3ar1-inhibited mice (Figures 6C–F, I,J). Because rhodopsin is a key protein in the phototransduction process, we analyzed the expression levels of rhodopsin and recoverin, revealing that they were significantly downregulated in wild-type mice compared to C3-deficient and C3ar1-inhibited mice after NaIO_3_ injection (Figures 6A,B,G,H).

### Involvement of the C3aR-STAT3 signaling pathway in retinal degeneration as determined by ribonucleic acid-seq

**FIGURE 7:**
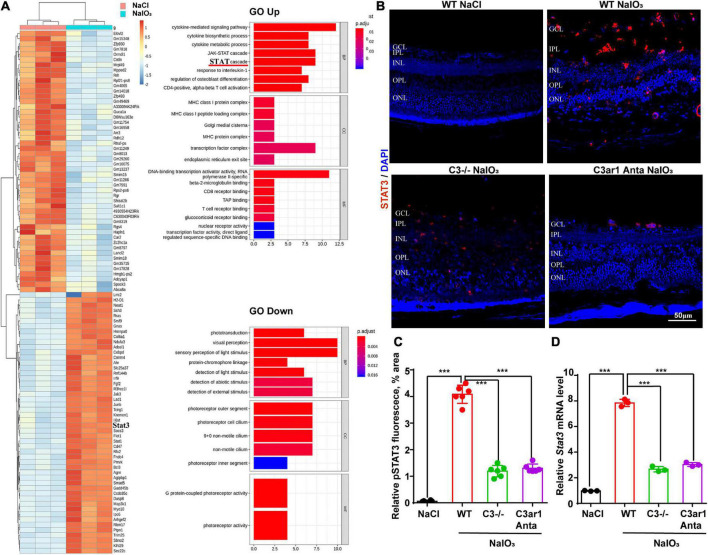
RNA-seq analysis revealed C3/C3aR/STA3 signaling pathway activation in the NaIO_3_ mouse model. **(A)** The top 100 DEGs in wild-type mice at 0 and 7 days after NaIO_3_ induction. Gene Ontology analysis of the signaling pathways related to up- and downregulated genes. **(B)** Immunofluorescence staining of STAT3 in the retinas of saline control- and NaIO3-treated WT, C3-KO and C3ar1-inhibited mice. **(C)** Quantification of STAT3 in the retinas of saline control- and NaIO3-treated WT, C3-KO and C3ar1-inhibited mice. **(D)** Stat3 mRNA expression in retinas as determined by qRT–PCR. Each bar corresponds to the mean ± *SD*. ***p < 0.001.

To further investigate the downstream mechanisms involving the C3a-C3ar1 pathway in photoreceptor degeneration, we compared the mRNA levels in NaIO_3_-treated retinas on day 7 with those in control retinas by RNA-seq analysis, revealing DEGs between control and NaIO_3_-treated retinas on day 7 (DE; fold-change > 2.0, *p* < 0.05). The downregulated DEGs included phototransduction genes, such as Arrestin3, Rhodopsin, and Rgr, while the C3, C3aR and STAT3 mRNA levels were obviously upregulated. GO enrichment analysis showed that the upregulated DEGs were involved in signaling pathways such as the STAT cascade, while the downregulated DEGs were involved in signaling pathways associated with phototransduction and visual perception (Figure 7A). A previous study indicated that C3ar1 regulates the expression of STAT3 and mediates inflammation during neuropathy (Litvinchuk et al., 2018), which is not clear in the retina. Real-time PCR and immunostaining confirmed the upregulation of STAT3 at both the mRNA and protein levels on day 7 after NaIO_3_ induction (Figures 7B–D). Knockout of C3 and C3ar1 inhibition also normalized the upregulation of STAT3. Our findings suggest that the enhanced expression of STAT3 contributes to microglial activation, the inflammatory response and photoreceptor degeneration after NaIO_3_ induction.

### Inhibition of STAT3 attenuates microglial activation and rescues visual function

**FIGURE 8:**
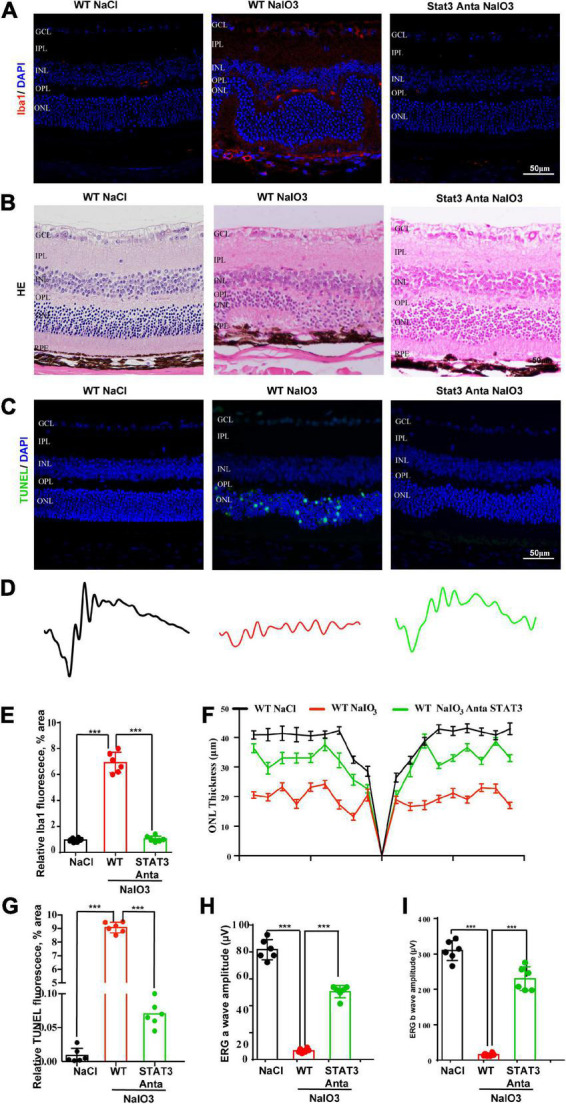
A pSTAT3 inhibitor inhibits microglial activation and rescues the retinal function of wild-type mice after NaIO_3_ induction. **(A)** Immunostaining of Iba in the retinas of saline control- and NaIO_3_-treated WT and pSTAT3-inhibited mice. **(B)** H&E staining of the retinas of saline control- and NaIO_3_-treated WT and pSTAT3-inhibited mice. **(C)** TUNEL immunofluorescence staining of retinas of saline control- and NaIO_3_-treated WT and pSTAT3-inhibited mice. **(D)** ERG analysis of retinal function in saline control- and NaIO_3_-treated WT and pSTAT3-inhibited mice. **(E)** Quantification of Iba1 in saline control- and NaIO_3_-treated WT and pSTAT3-inhibited mice. **(F)** Quantification of ONL thickness in the retinas of saline control- and NaIO_3_-treated WT and pSTAT3-inhibited mice. **(G)** Quantification of TUNEL in retinas of saline control- and NaIO_3_-treated WT and pSTAT3-inhibited mice. **(H,I)** Bar plot of the amplitudes of a and b waves in saline control- and NaIO_3_-treated WT and pSTAT3-inhibited mice. ***p < 0.001.

Previous studies have shown that the activation of the STAT pathway is involved in the immune response (Litvinchuk et al., 2018; Reichenbach et al., 2019). To better understand the role of the C3a/C3aR/STAT3 signaling pathway in photoreceptor degeneration, we pharmacologically inactivated STAT3 using the specific inhibitor SH-4-54. WT mice were pretreated with the STAT3 inhibitor SH-4-54 (10 mg/kg) 3 days before the application of NaIO_3_. Fluorescence immunostaining revealed that SH-4-54 obviously downregulated microglial cell activation. The Iba1-positive ratio of microglial cells was alleviated after the application of SH-4-54 (Figures 8A,E). The H&E staining results revealed the protective effect of the ONL layer, and the ERG results showed that the a-wave and b-wave amplitudes were partially preserved, indicating the retinal protective effect of the STAT3 inhibitor (Figures 8B,D,F,H,I). Furthermore, TUNEL staining showed that the apoptosis of photoreceptor cells was significantly reduced after the application of SH-4-54 (Figures 8C,G). Overall, these results established a novel signaling pathway (C3/C3aR/STAT3) linking microglial activation and photoreceptor cell degeneration to oxidative stress conditions.

## Discussion

The mechanism underlying retinal degeneration has not been fully elucidated, which severely restricts the development of new therapies. Strong evidence supports a key role of complement and the neuroinflammatory response in retinal diseases. Understanding the detailed mechanism will facilitate the development of potential treatments for photoreceptor cell degeneration-related diseases. In this study, we revealed that the C3a/C3aR/STAT3 pathways are important for mediating the immune response and photoreceptor cell apoptosis under oxidative stress conditions, which might be helpful for treating retinal degeneration diseases.

Complement and microglial cells mediate neural cell damage in many neural degeneration diseases, such as age-related macular degeneration (AMD), Stargard’s disease and glaucoma (Jha et al., 2011; Lenis et al., 2017; Natoli et al., 2017). One previous study showed that irreversible photoreceptor cell damage in mice treated with NaIO_3_ is related to macrophage accumulation (Moriguchi et al., 2018), and oxidative stress and complement activation have been correlated in AMD models (Pujol-Lereis et al., 2016). As the critical component of the complement system, C3 plays a role in photoreceptor cell death, but the mechanism is not clear. In this study, we observed the activation of microglial cells at 7 days after NaIO_3_ injection, and the colocalization of C3 and microglial cells was consistent with previous reports. We also detected the intracellular C3 expression in retinal pigment epithelium (RPE) cells at 1 day after NaIO_3_ injection (Supplementary Figure 2). Furthermore, RNA-seq data revealed the upregulated expression of C3ar1 and other components under oxidative stress conditions. This inspired us to explore the role of C3a-C3ar1 in retinal injury induced by NaIO_3_. Simultaneously, Pauly et al. (2019) were the first to explore the characteristics of complement components in specific retinal cell types under normal and pathological conditions using single-cell RNA-seq analysis. Their results strongly indicate that a local retinal complement that is independent of the systemic components typically produced by the liver is activated during retinal degeneration. For the first time, we found that NaIO_3_-induced photoreceptor apoptosis depended on C3a-C3ar1 axis activation and was partially mediated by the knockout of C3 or inhibition of C3ar1. However, the characteristics of macrophages in the peripheral infiltrating blood in the retina are not clear. One previous study found that the complement C3a signaling pathway mediates peripheral infiltrating blood monocytes and then facilitates skeletal regeneration (Zhang et al., 2017). Thus, we need to fully demonstrate the role of C3a-C3ar1 activation in macrophage infiltration and activation in the future.

Mechanistically, we identified STAT3 as a downstream gene of the C3/C3aR pathway during photoreceptor cell degeneration. Our RNA-seq analysis results revealed significant activation of the C3/C3aR/STAT cascade in the NaIO_3_-induced retinal degeneration model. Strikingly, genetic deletion of C3 and pharmacological inhibition of C3aR or STAT3 led to the prevention of photoreceptor loss and preservation of retinal function. These findings are consistent with the previously reported critical role of C3aR in mediating CNS immune homeostasis and tau pathology by targeting a transcription factor network in human AD (Litvinchuk et al., 2018). Several studies have found that STATA3 is involved in RPE cell survival, the inflammatory response, visual cycle maintenance and cytokine release (Patel and Hackam, 2013; Patel et al., 2013). Moreover, features of the immune response, such as proinflammatory cytokine release, microglial infiltration and Muller glial reactivity, were suppressed after the C3/C3aR/STAT3 pathway was blocked in our study. Additionally, given the complexity and the degree of crosstalk between various immune pathways, other transcription factors (TFs), such as Hmox1, NFkB, and NFE2L2, might also interact with the C3a-C3ar1 pathway and be involved in retinal degeneration (Karlen et al., 2020; Potilinski et al., 2021).

Taken together, the results of this study demonstrate that the activation of the C3a/C3aR/STAT3 pathway plays an important role in mediating microglial activation and photoreceptor cell degeneration. Therefore, inhibition of local C3 and normalization of the C3a/C3aR/STAT3 pathway could be helpful for the prevention of AMD and other retinal degeneration diseases.

## Data availability statement

The original contributions presented in the study are publicly available. This data can be found here: The RNA-seq data are available at: https://ngdc.cncb.ac.cn/search/?dbId=&q=CRA007710.

## Ethics statement

The animal study was reviewed and approved by Institutional Animal Care and Use Committee of the General Hospital of the Chinese People’s Liberation Army and Zhengzhou University. All experiments were performed in accordance with the National Institutes of Health Guidelines for the Care and Use of Laboratory Animals (Id number: 2020-ky-67).

## Author contributions

SW and LD conceived and designed the study, constructed the animal, and performed the RPE cell culture and immunohistochemistry experiments. SW, LD, and SY analyzed the data and wrote the manuscript. G-HP revised the manuscript, provided support, and supervised the project. All authors read and approved the final manuscript.

## Supplementary material

Supplementary Figure 1 Schematic of the retina with a sample location.

Supplementary Figure 2 Intracellular C3 activation in RPE cells after NaIO_3_ injury *in vivo*. **(A)** Double immunofluorescent staining C3 (green) and RPE65 (red) of wild-type 0d and 1d after NaIO_3_ injection. **(B)** Confocal assay of C3 localization of wild-type 0d and 1d after NaIO_3_ injection. **(C)** TUNEL immunofluorescent staining of wild-type and C3-deficient mice 1d after NaIO_3_ injection. **(D)** Quantification of **(C)**.
