# Supplementary figures and images for: Complement C3a receptor inactivation attenuates retinal degeneration induced by oxidative damage
Source: Front Neurosci. 2022 Aug 30;16:951491. doi: 10.3389/fnins.2022.951491 (PMC9469738; doi:10.3389/fnins.2022.951491)

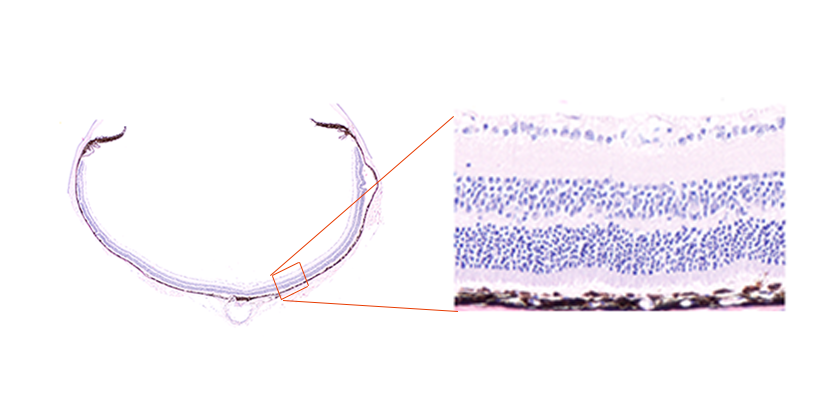

Supplement: Supplementary Figure 1 — Schematic of the retina with a sample location. [file Image_1.TIF]

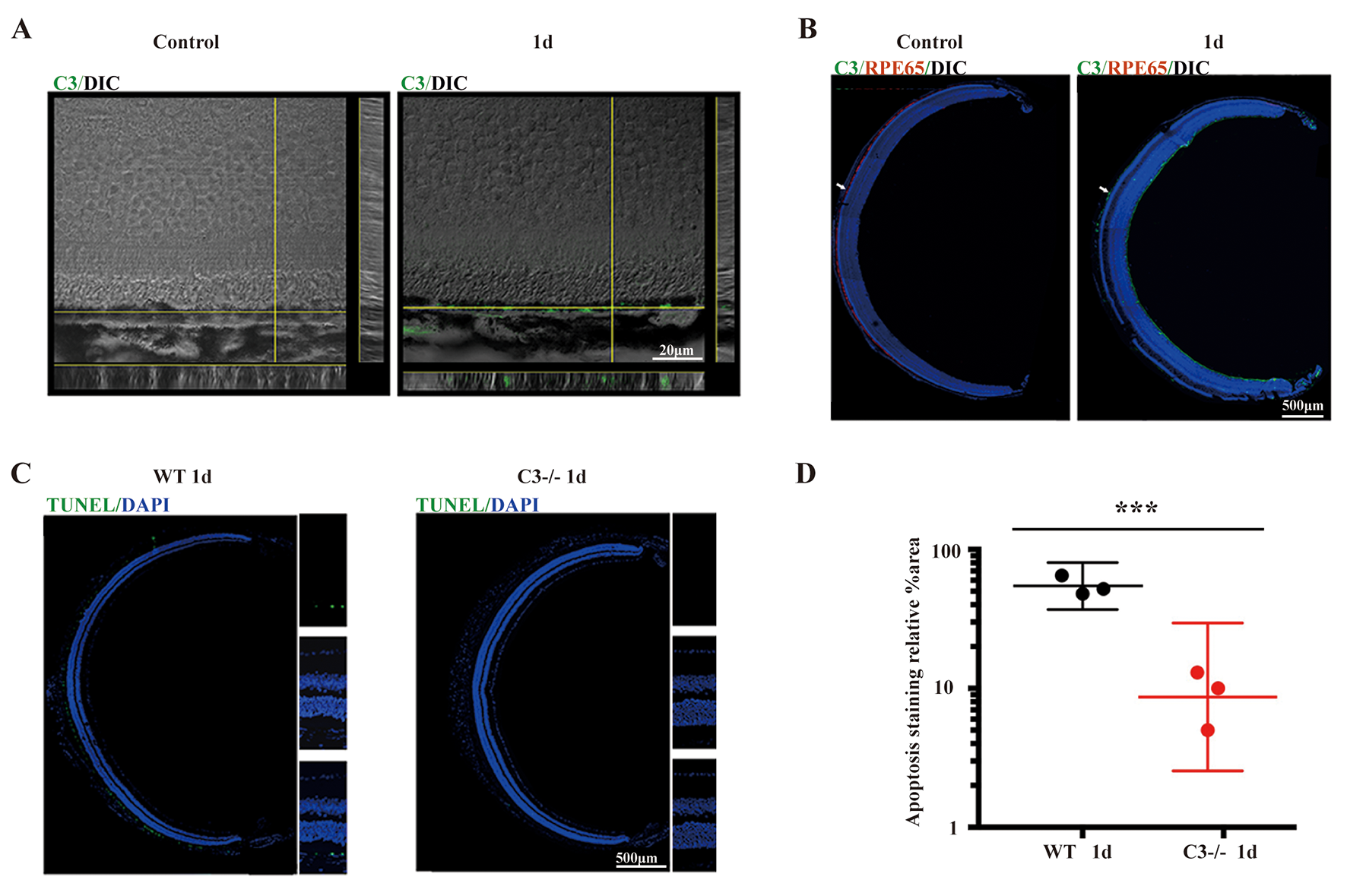

Supplement: Supplementary Figure 2 — Intracellular C3 activation in RPE cells after NaIO3 injury in vivo. (A) Double immunofluorescent staining C3 (green) and RPE65 (red) of wild-type 0d and 1d after NaIO3 injection. (B) Confocal assay of C3 localization of wild-type 0d and 1d after NaIO3 injection. (C) TUNEL immunofluorescent staining of wild-type and C3-deficient mice 1d after NaIO3 injection. (D) Quantification of (C). [file Image_2.TIF]
